# Effective adoptive immunotherapy of triple-negative breast cancer by folate receptor-alpha redirected CAR T cells is influenced by surface antigen expression level

**DOI:** 10.1186/s13045-016-0285-y

**Published:** 2016-07-20

**Authors:** De-Gang Song, Qunrui Ye, Mathilde Poussin, Jessica A. Chacon, Mariangela Figini, Daniel J. Powell

**Affiliations:** Ovarian Cancer Research Center, Department of Obstetrics and Gynecology, Perelman School of Medicine, University of Pennsylvania, 3400 Civic Center Blvd, Rm 8-103 Smilow CTR, Philadelphia, PA 19104 USA; Department of Experimental Oncology and Molecular Medicine, Fondazione IRCCS Istituto Nazionale dei Tumori, Milan, Italy; Department of Pathology and Laboratory Medicine, Abramson Cancer Center, Perelman School of Medicine, University of Pennsylvania, 3400 Civic Center Blvd, Rm 8-103 Smilow CTR, Philadelphia, PA 19104 USA; Current address: Intrexon Corporation, 20358 Seneca Meadows Pkwy, Germantown, MD 20876 USA

**Keywords:** Folate receptor-alpha, Triple-negative breast cancer, Chimeric antigen receptor, Immunotherapy

## Abstract

**Background:**

The poor prognosis and the limited efficacy of targeted therapy in patients with triple-negative breast cancer (TNBC) have raised the need for alternative therapies. Recent studies have demonstrated that folate receptor-alpha (FRα) may represent an ideal tumor-associated marker for immunotherapy for TNBC.

**Methods:**

The aim of the present study was to apply a chimeric antigen receptor (CAR) approach for the targeting of FRα expressed on TNBC cells and evaluate the antitumor activity of CAR-engineered T cells in vitro and in vivo.

**Results:**

We found that human T cells expressing a FRα-specific CAR were potent and specific killers of TNBC cells that express moderate levels of FRα in vitro and significantly inhibited tumor outgrowth following infusion into immunodeficient mice bearing an MDA-MB-231 tumor xenograft. However, the antitumor activity of the FRα CAR T cells was modest when compared to the same CAR T cells applied in an ovarian tumor xenograft model where FRα expression is more abundant. Notably, FRα CAR T cells induced superior tumor regression in vivo against MDA-MB-231 that was engineered for overexpression of FRα.

**Conclusions:**

Taken together, our results show that FRα CAR T cells can mediate antitumor activity against established TNBC tumor, particularly when FRα is expressed at higher levels. These results have significant implications for the pre-selection of patients with high antigen expression levels when utilizing CAR-based adoptive T cell therapies of cancer in future clinical trials.

**Electronic supplementary material:**

The online version of this article (doi:10.1186/s13045-016-0285-y) contains supplementary material, which is available to authorized users.

## Background

Triple-negative breast cancer (TNBC) is characterized by the limited expression of the human epidermal growth factor receptor 2 (HER2), estrogen receptor (ER), and progesterone receptor (PR) and accounts for approximately 15 % of invasive breast cancers. Patients with TNBC do not benefit from HER2-based targeted therapy or endocrine therapy [1]. Chemotherapy is currently the mainstay of systemic medical treatment, although patients with TNBC have a worse outcome after chemotherapy compared to breast cancer patients with other subtypes [2], a finding that reflects the intrinsically adverse prognosis associated with the disease. Thus, new and powerful therapies are urgently needed for TNBC patients.

A number of potential antigen targets have been validated in TNBC [3]. Folate receptor-alpha (FRα) is highly expressed in non-mucinous tumors of epithelial origin including ovarian, breast, and lung cancers and expressed at low levels on the apical surface of a subset of polarized epithelial cells including the parotid, kidney, lung, thyroid, and breast. Specific overexpression of FRα in certain malignancies, including TNBC [4], with low coordinate expression in normal tissue, makes FRα an attractive target for directed therapies. In breast cancer, FRα expression can be regulated by steroid hormones, particularly estrogens [5, 6]. Specifically, 17β-estradiol has been demonstrated to down-regulate FRα expression by direct action of the ER on the P4 promoter of FRα, suggesting a negative correlation between the expression of ER and FRα. Indeed, O’Shannessy et al. demonstrated that ER-negative breast cancer samples were significantly more likely to express FRα than ER-positive cancers. Taken together, these findings rationalize the assessment of FRα as a tumor-associated antigen candidate for the targeted therapy of TNBC.

Given its cancer-centric overexpression, FRα has been an attractive candidate for targeted drug delivery using folate-conjugated therapeutic compounds that bind FRα or murine, chimeric, and humanized monoclonal antibodies (mAbs) alone or in conjugates to deliver radionuclides, T cells, and stimulatory cytokines to malignant tissue [7, 8]. Additionally, the transfer of T cells genetically redirected with a chimeric antigen receptor (CAR) specific for FRα is an attractive technology that is actively being investigated [9, 10]. The CAR approach combines the antigen specificity of an antibody with the ability of T cells to mediate the killing of tumor cells in a single fusion molecule. CAR-modified T cells actively and specifically target their specified antigen and have the capacity to persist as memory cells in vivo [9, 10]. As such, CAR-modified T cells that target tumor-associated antigens (TAAs), such as FRα, may be more effective than mAbs in generating durable tumor responses. Here, we generated a FRα-specific CAR with an intracellular CD27 co-stimulatory signaling domain and evaluated the therapeutic efficacy of T cells transduced to express this CAR in a murine xenograft model of human TNBC. We demonstrate that FRα-specific CAR T cells have the capacity to inhibit human TNBC growth in vivo and that more robust tumor regression is achievable when the TNBC cells overexpress surface FRα protein.

## Methods

### Cell lines

Lentivirus packaging was executed using the immortalized normal fetal renal 293T cell line purchased from ATCC. Human cell lines used in immune-based assays include the established human ovarian cancer cell lines SKOV3 and C30 and breast cancer cell lines T47D, SKBR3, MCF7, MDA-231, MDA-436, MDA-468, MDA-453, and BT549. For bioluminescence assays, the cancer cell lines were transfected to express firefly luciferase (fLuc). The mouse malignant mesothelioma cell line, AE17 (kindly provided by Steven Albelda, University of Pennsylvania), was used as antigen negative control. All cell lines were maintained in R10 medium: RPMI-1640 supplemented with 10 % heat-inactivated FBS, 100 U/mL penicillin, 100 mg/mL streptomycin sulfate, and 10 mmol/L HEPES.

### CAR construction and lentivirus production

**Fig. 1:**
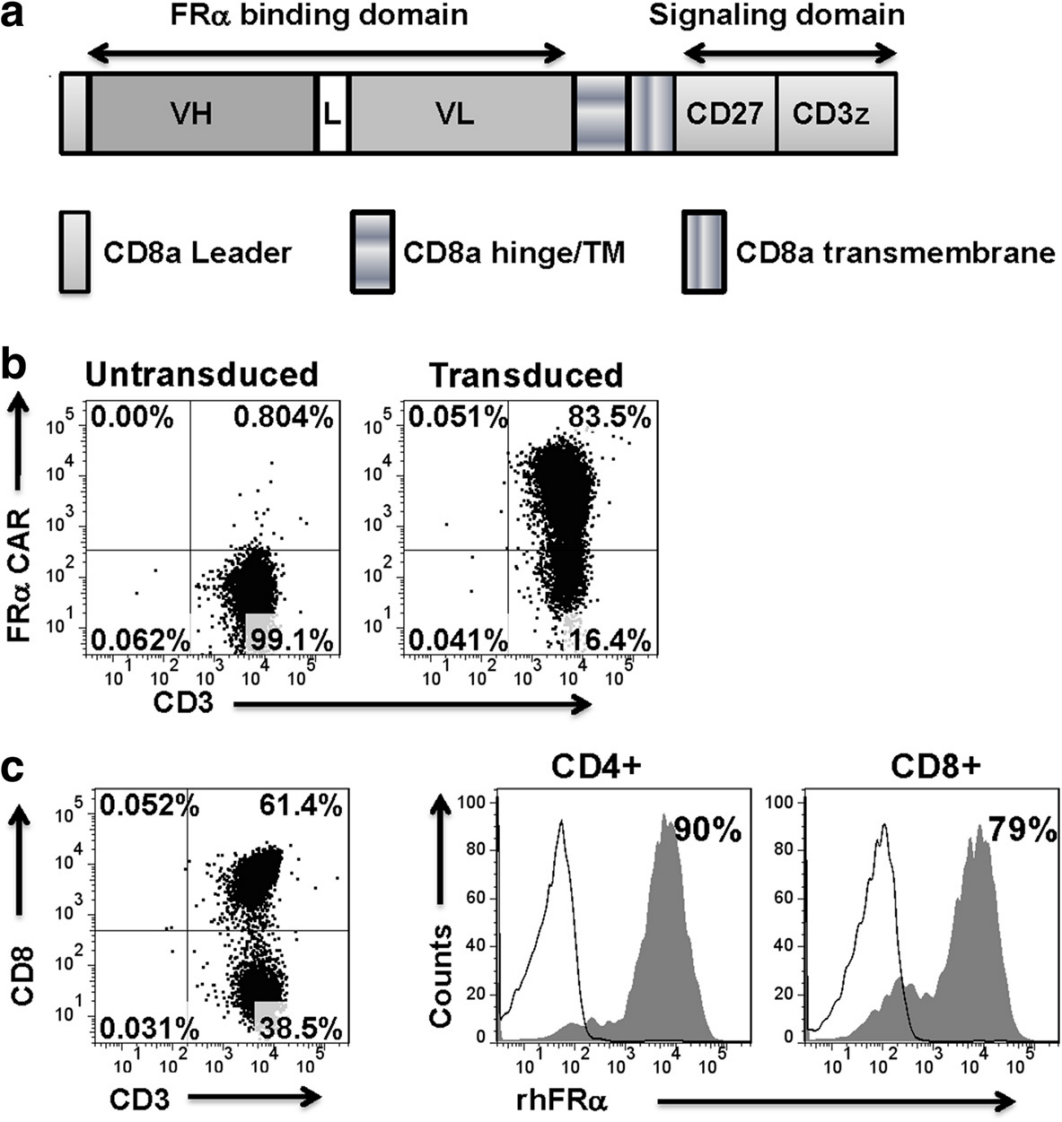
Construction and expression of folate receptor-alpha (FRα)-specific chimeric antigen receptor (CAR). **a** Schematic representation of MOv19-based FRα CAR constructs containing the CD27 co-stimulatory module in combination with the CD3ζ cytosolic domain. **b** Primary human CD3 T cells can efficiently express FRα-specific CAR. Expression was detected via PE-conjugated goat anti-mouse F(ab′)_2_ fragment and measured by flow cytometry. **c** Compared to untransduced (UNT) T cells, transduced T cells consisted of CD4- and CD8-positive cells with both subsets expressing FRα CAR. FRα CAR expression was detected via biotin-labeled recombinant FRα protein staining followed by streptavidin-PE after transduction with lentivirus. Transduction efficiencies are indicated with the percentage of CAR expression in parentheses

The anti-FRα CAR construct was comprised of the MOv19 scFv linked to a CD8a hinge and transmembrane region, followed by a CD3z signaling moiety in tandem with the CD27 intracellular signaling motif (MOv19-27z; Fig. 1a) and was previously described [9, 10]. An anti-CD19 CAR containing CD3z and CD27 signaling motifs in tandem (CD19-27z) was used as an antigen specificity control [9, 11]. High-titer replication-defective lentiviruses were produced and concentrated as previously described [12]. Briefly, 293T cells were seeded in 150-cm^2^ flask and transfected using Express In (Open Biosystems) according to the manufacturer’s instructions. FRα-specific CAR transgene plasmids (15 μg) were co-transfected with 18 μg pRSV.REV (Rev expression plasmid), 18 μg pMDLg/p.RRE (Gag/Pol expression plasmid), and 7 μg pVSV-G (VSV glycoprotein expression plasmid) with 174 μL Express In (1 μg/μL) per flask. Supernatants were collected at 24 and 48 h after transfection, concentrated 10-fold by ultracentrifugation for 2 h at 28,000 rpm with a Beckman SW32Ti rotor (Beckman Coulter). The viruses were aliquoted into tubes and stored at −80 °C until ready to use for titering or experiments. All lentiviruses used in the experiments were from concentrated stocks.

### Human T cells and transfection

Primary human T cells, purchased from the Human Immunology Core at University of Pennsylvania, were isolated from healthy, normal donors following leukapheresis by negative selection. All T cell samples were collected under a protocol approved by a University Institutional Review Board, and written informed consent was obtained from each healthy, normal donor. T cells were cultured in R10 medium and stimulated with anti-CD3 and anti-CD28 monoclonal antibody (mAb)-coated beads (Invitrogen). Approximately 18 to 24 h after activation, human T cells were transduced using a spinoculation procedure. Briefly, 0.5 × 10^6^ T cells were infected with a multiplicity of infection (MOI) of 5 of the MOv19-27z vector. A mixture of cells and vectors were centrifuged at room temperature for 90 min (2500 rpm) in a table-top centrifuge (Sorvall ST 40). After the engineered T cells were rested, as determined by decreased growth kinetics and cell size which is measured using the Multisizer 3 Coulter Counter (Beckman Coulter), the rested T cells were then used for functional analysis.

### Flow cytometric analysis

The following fluorochrome-conjugated mAbs, purchased from BD Biosciences, were used for phenotypic analysis: APC-Cy7 anti-human CD3, fluorescein isothiocyanate (FITC) anti-human CD4, APC anti-human CD8, PE-anti-human CD45, and PE anti-human CD137. 7-Aminoactinomycin D (7-AAD) was used for viability staining. For the in vivo mouse T cell transfer experiments, peripheral blood was obtained via retro-orbital bleeding and stained for the presence of human CD45, CD4, and CD8 T cells. Gating specifically on the human CD45+ population, the CD4+ and CD8+ subsets were quantified using TruCount tubes (BD Biosciences) with known numbers of fluorescent beads as described in the manufacturer’s instructions. Tumor cell lines and enzymatically digested MDA-231 tumor cell surface expression of FRα was measured using human PE-FRα mAb (R&D Systems, Inc.) and Quanti-Brite PE beads (BD Bioscience). The FRα PE-conjugated antibody was used at a 1:1 PE/protein ratio for the quantitative analysis of surface FRα expression. FRα antigen copy number per tumor cell was evaluated by comparing the mean fluorescence intensity (MFI) versus the number of known PE molecules per bead. A calibration curve was constructed per cell line to calculate the mean number of PE molecules per cell for the FRα+ cell population. Mouse mesothelioma cell line AE17 was used as a negative control, and the background human FRα receptors/cell values measured on AE17 were subtracted from the FRα receptors/cell values detected on breast cancer cell lines.

FRα CAR surface expression was evaluated using recombinant FRα-Fc protein (R&D Systems) followed by PE-labeled anti-human IgG Fc gamma-specific antibody (eBioscience) or biotin-labeled goat anti-mouse IgG F(ab′)_2_ fragment followed by streptavidin-APC. For intracellular cytokine staining, T cells were stimulated in R10 media containing phosphomolybdic acid (PMA) (30 ng/mL) (Sigma-Aldrich), ionomycin (500 ng/mL) (Sigma-Aldrich), and monensin (GolgiStop) (1 μL/mL) (BD Biosciences) in a cell incubator with 10 % CO_2_ at 37 °C for 4 h. Cytokine production in CAR T cells was determined by co-culturing CAR T cells with FRα^pos^ ovarian cancer cells for 5 h. After the cell surface markers were stained, the cells were then fixed and permeabilized using Cytofix/Cytoperm and Perm/Wash buffer (eBioscience) according to the manufacturer’s instructions. The cells were then stained with the following fluorescence-conjugated cytokine antibodies: PE anti-human interferon-gamma (IFN-γ), Pacific Blue anti-human TNF-α, and FITC anti-human IL-2. All flow cytometry was conducted using a BD FACSCanto II flow cytometer (BD Biosciences), and flow cytometric data were analyzed with FlowJo version 7.6.1 software (Tree Star, Ashland, OR).

### Cytokine release assays

Cytokine release assays were performed by co-culturing 1 × 10^5^ T cells with 1 × 10^5^ target cells in triplicate in a 96-well flat bottom plate in a total volume of 200 μL R10 media. After 20–24 h, co-culture supernatants were collected and ELISA (Biolegend, San Diego) was performed, according to the manufacturer’s instructions, to measure the secretion of IFN-γ. The values shown represent the mean of triplicate wells.

### Cytotoxicity assays

For cell-based bioluminescence assays, 5 × 10^4^ firefly luciferase (fLuc)-expressing tumor cells were cultured with R10 media in the presence of different T cell ratios in a 96-well Microplate (BD Biosciences). After incubation for ~20 h at 37 °C, each well was filled with 50 μL of DPBS resuspended with 1 μL of d-luciferin (0.015 g/mL) and imaged with the Xenogen IVIS Spectrum. Tumor cell viability percentage was calculated as the mean luminescence of the experimental sample minus background divided by the mean luminescence of the input number of target cells used in the assay minus background times 100. All data are represented as a mean of triplicate wells.

### Xenograft model of TNBC and ovarian cancer

All animals were obtained from the Stem Cell and Xenograft Core of the Abramson Cancer Center, University of Pennsylvania. NOD/SCID/γ-chain^−/−^ (NSG) mice (6–12 weeks old) were bred, treated, and maintained under pathogen-free conditions in-house under University of Pennsylvania IACUC-approved protocols. To establish a TNBC model, 6- to 12-week-old female NSG mice were inoculated subcutaneously (s.c.) on the flank with 3 × 10^6^ MDA-231 fLuc+ or MDA-231.FR fLuc cells on day 0. For the ovarian cancer model, NSG mice were inoculated s.c. on the flank with 5 × 10^6^ SKOV3 fLuc+ cells. After the tumors become palpable at about 3 weeks, primary human T cells were activated and transduced as described above. After the primary human T cells were expanded for 2 weeks and the mouse tumor burden was about 200–300 mm^3^, the mice were treated with the T cells. The route, dose, and timing of T cell injections are indicated in the individual figure legends. Tumor dimensions were measured with calipers and tumor volumes calculated using the formula *V* = 1/2(length × width^2^), where length is the greatest longitudinal diameter and width is the greatest transverse diameter. Animals were imaged prior to T cell transfer and about every week thereafter to evaluate tumor growth. Photon emission from fLuc+ cells was quantified using the “Living Image” software (Xenogen) for all in vivo experiments. Approximately 40 days after the first T cell injection, the mice were euthanized and the tumors were resected immediately in order to calculate the tumor volumes.

### Bioluminescence imaging

Tumor growth was also monitored using bioluminescence imaging (BLI). BLI was conducted using Xenogen IVIS imaging system. The photons emitted from fLuc-expressing cells within the animal body were quantified using Living Image software (Xenogen). Briefly, mice bearing MDA-231 fLuc+ or MDA-231.FR fLuc+ tumor cells were injected intraperitoneally (i.p.) with d-luciferin (150 mg/kg stock, 100 μL of d-luciferin per 10 g of mouse body weight) suspended in PBS and imaged under isoflurane anesthesia after 5~10 min. A pseudocolor image representing light intensity (blue, least intense; red, most intense) was generated using Living Image. BLI findings were confirmed at necropsy.

### Statistical analysis

The data are reported as means and standard deviations (SDs). Statistical analysis was performed using two-way repeated-measure analysis of variance (ANOVA) for the tumor burden (tumor volume, photon counts). Student’s *t* test was used to evaluate differences in absolute numbers of transferred T cells, cytokine secretion, and specific cytolysis. GraphPad Prism 5.0 (GraphPad Software) was used for the statistical calculations, where a *p* value of *p* < 0.05 was considered significant.

## Results

### Construction and expression of FRα-specific CAR

A FRα-specific CAR-encoding lentivirus construct was generated comprised of the anti-human FRα-specific MOv19 scFv [13] linked to a CD8α hinge and transmembrane region, followed by a CD27 intracellular signaling motif in tandem with the CD3ζ signaling moiety, and referred to as MOv19-27z (Fig. 1a). Primary human CD3+ T cells were efficiently transduced with CAR lentiviral vectors with reproducible transduction efficiencies of ~80 % (Fig. 1b). Surface expression of the FRα CAR on CD4+ and CD8+ T cells was detectable using recombinant FRα protein staining (Fig. 1c), demonstrating positive CAR expression and antigen binding capacity.

### FRα is expressed on the surface of TNBC cell lines

**Fig. 2:**
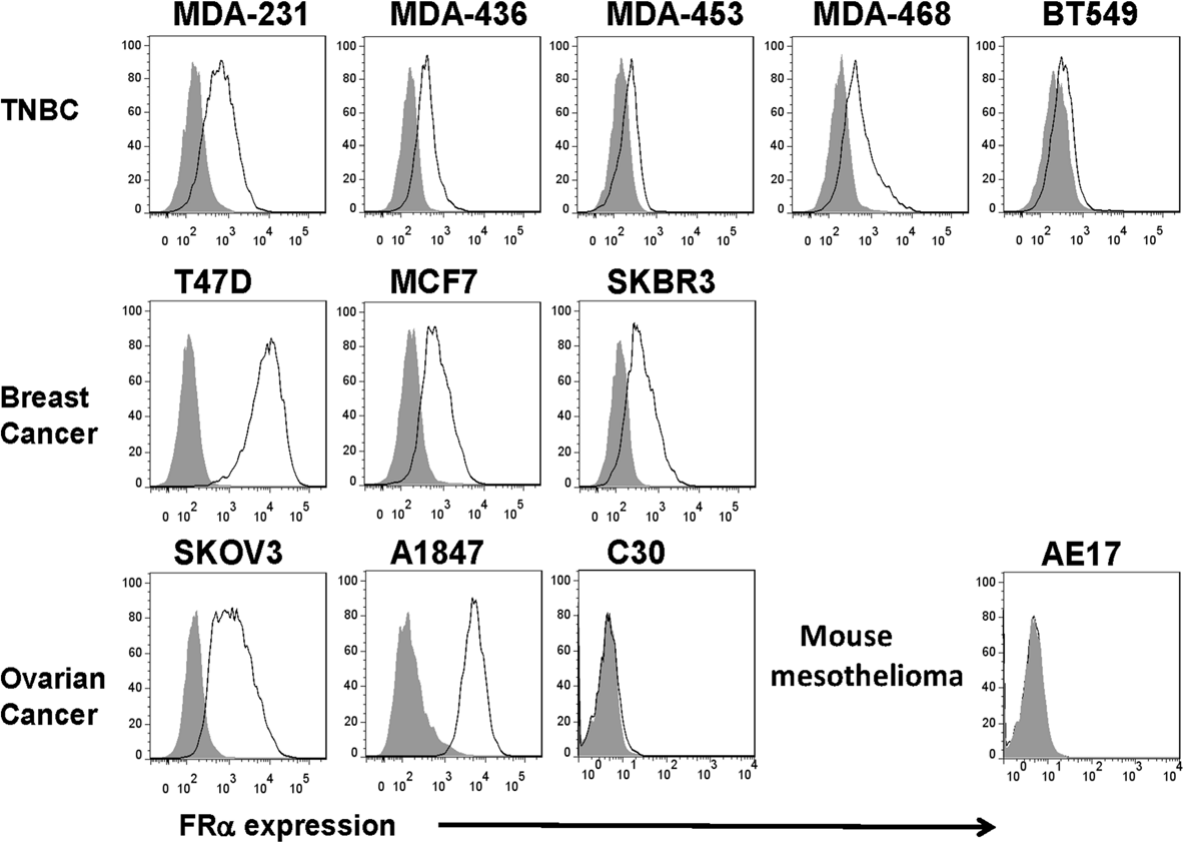
FRα may represent a new therapeutic target in TNBC. FRα surface expression in TNBC cell lines was measured by flow cytometry. FRα-specific mAb MOv18 was used to measure FRα expression on various ovarian cancer (OC) and breast cancer (BC) cell lines (*open empty histogram*), compared to a matched isotype antibody control (*filled gray histogram*)

Using flow cytometry, surface expression of FRα protein was determined on a variety of tumor cell lines after staining the cells with anti-FRα antibody. FRα expression was detected in ovarian cancer lines SKOV3 and A1847 at high levels and also on the breast cancer cell lines, T47D, SKBR3, and MCF7; the C30 ovarian cancer cell line served as an antigen negative control (Fig. 2). Importantly, all five TNBC cell lines tested, including MDA-231, MDA-468, MDA-436, MDA-453, and BT549, expressed FRα protein on their cell surface at moderate to low levels (Fig. 2). As demonstrated in Additional file 1: Figure S1A, there was a range of FRα molecules expressed per cell among the breast cancer cell lines tested.

### FRα CAR T cells specifically recognize FRα^pos^ TNBC cell lines in vitro

**Fig. 3:**
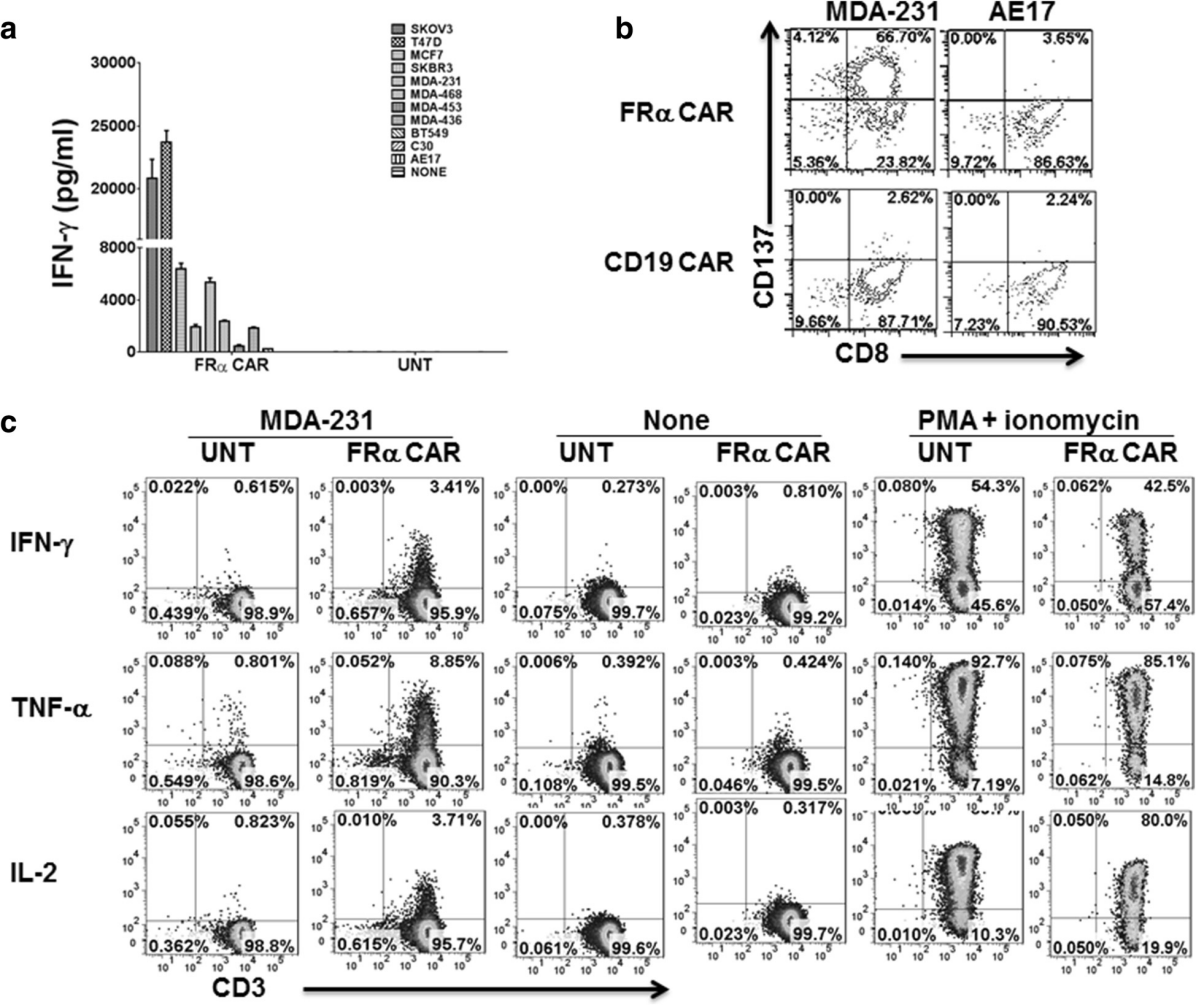
FRα CAR T cells secrete Th1 cytokines and up-regulate CD137 in response to FRα (+) tumor cells. **a** IFN-γ secretion of FRα CAR-transduced T cells after 20 h co-culture with the indicated tumor lines at a 1:1 ratio. Untransduced (UNT) T cells were used as a negative control. **b** Antigen-specific T cell activation was detected by the induction of CD137 expression. **c** FRα CAR T cells were stimulated with MDA-231 cells for 5 h in the presence of *Golgi* stop and analyzed by flow cytometry for intracellular cytokines IFN-γ, TNF-α, and IL-2. UNT T cells served as our negative control, whereas PMA and ionomycin-treated T cells served as positive controls

To determine whether human FRα CAR-modified T cells were able to recognize and react against FRα^pos^ TNBC cells, FRα CAR-bearing T cells were co-cultured overnight with the TNBC cell lines MDA-231, MDA-468, MDA-436, MDA-453, or BT549, and IFN-γ secretion in the cell culture supernatants was measured by ELISA. Since ovarian cancers and breast cancers frequently express FRα, established human ovarian cancer cell lines (SKOV3) and breast cancer cell lines (T47D, SKBR3, and MCF7) that express surface FRα at varying levels were used for positive control targets, while FRα-negative cell lines C30 and AE17 were used as negative controls. As shown in Fig. 3a, FRα CAR T cells secreted substantial amounts of IFN-γ, denoting T cell activation after co-culture with FRα^pos^ TNBC cell lines. The number of FRα molecules/cells correlated with the amount of IFN-γ secreted (Additional file 1: Figure S1B) and determined the coefficient of determination value (*R*^2^ = 0.62). No IFN-γ production was detected when FRα CAR T cells were cultured with FRα-negative targets (C30 and AE17) or from co-cultures containing untransduced (UNT) T cells. Up-regulated CD137 (4-1BB) expression represents a surrogate marker for antigen-specific activation of functional human CD8+ T cells [14]. When FRα CAR T cells were co-cultured with FRα^pos^ or FRα^neg^ tumor cells, robust up-regulation of CD137 by CAR T cells was observed only when incubated with FRα^pos^ TNBC cells (Fig. 3b). CD137 up-regulation was not detected on anti-CD19 CAR T cells when similarly cultured with FRα^pos^ TNBC cells, indicating that CD137 up-regulation by FRα CAR T cells was antigen-specific.

In addition to the above assays, representative fluorescence-activated cell sorter (FACS) plots of 5-h intracellular expression of proinflammatory cytokines by FRα CAR T cells in response to FRα^pos^ TNBC cells are shown (Fig. 3c). Th1 cytokines including IFN-γ, TNF-α, and IL-2 were exclusively expressed in FRα CAR T cells and not in UNT control T cells, when incubated with the FRα^pos^ MDA-231 TNBC cell line. PMA/ionomycin-treated T cells served as positive controls for T cell-stimulated cytokine production.

### FRα CAR T cells have antitumor activity against MDA-231 in vitro and in vivo

**Fig. 4:**
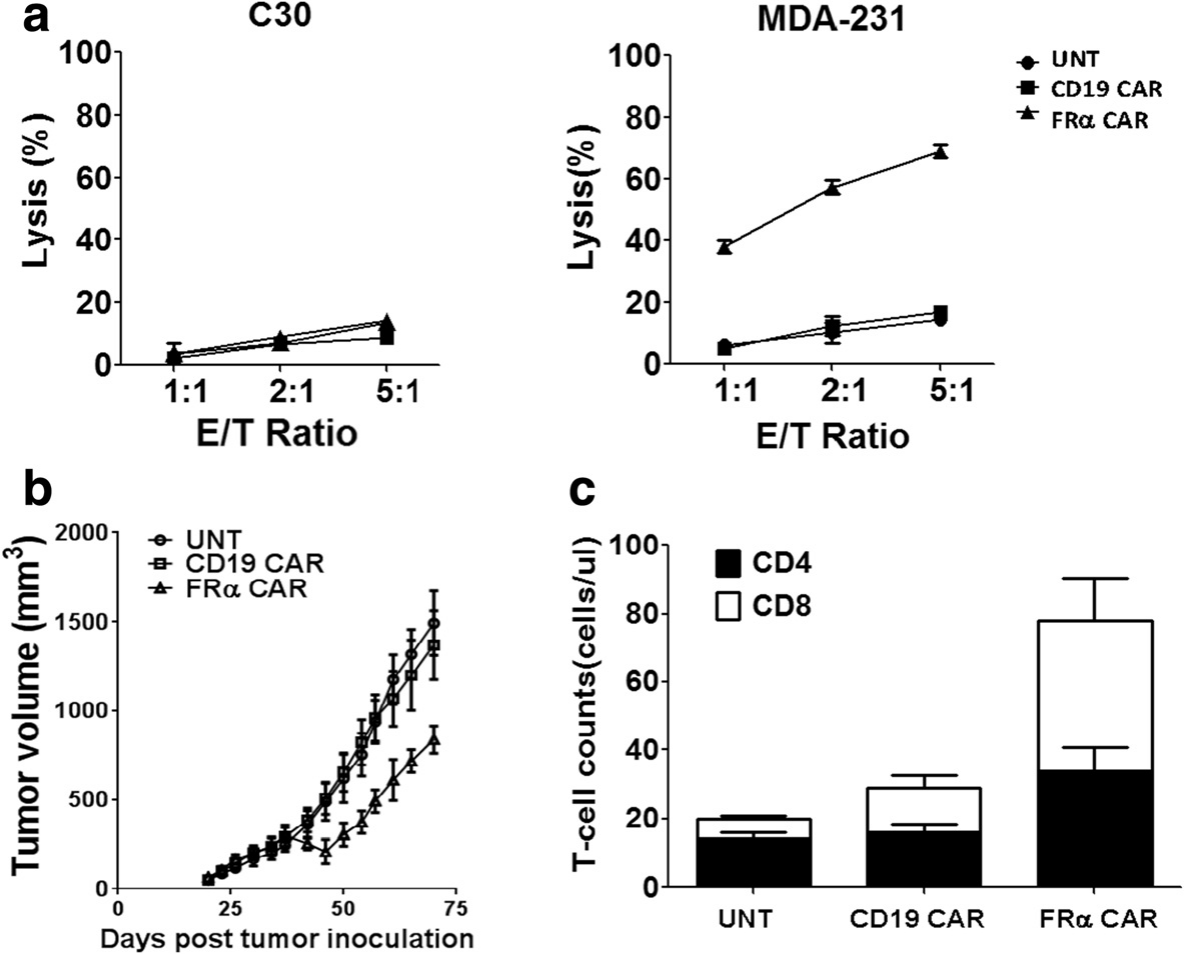
Anti-tumor activity of FRα CAR T cells in vitro and in vivo. **a** FRα CAR T cells lysed FRα+ MDA-231 cells but exhibited decreased lysis of the FRα-C30 cells at the indicated effector/target (E/T) ratio for ~20 h. Untransduced (UNT) T cells served as our negative control. **b** NSG mice bearing established subcutaneous (s.c.) tumor were treated with intravenous (i.v.) injections of 1 × 10^7^ CAR+ T cells on days 40 and 46 post tumor inoculation. Tumor growth was assessed by caliper measurement [V = 1/2(length × width^2^)]. **c** Peripheral blood was collected 3 weeks after the first T cell infusion and quantified for the absolute number of human CD4+ and CD8+ T cells/μL of blood. Mean cell count ± SEM is shown with *n* = 5 for all groups

The cytolytic activity of FRα CAR T cells in vitro was evaluated using an overnight bioluminescence assay (Fig. 4a). FRα CAR T cells had robust and specific cytotoxic activity against FRα^pos^ MDA-231 cells but not FRα-negative C30 cells. Untransduced or control anti-CD19 CAR T cells did not lyse MDA-231 or C30 cell lines.

To assess the antitumor activity of CAR T cells targeting FRα^pos^ tumor in vivo, we first evaluated the potency of FRα CAR T cells using a xenograft mouse model of TNBC tumor. Immunodeficient non-obese diabetic/severe combined immunodeficient/IL-2γc^null^ (NSG) mice received subcutaneous inoculation of firefly luciferase (fLuc+) FRα^pos^ human TNBC MDA-231 cells on the hind flank and received intravenous (I.V.) injections of 10^7^ CAR+ T cells on days 40 and 46 after tumor inoculation, when tumors were ~250 mm^3^ in size and evident by bioluminescence imaging (BLI). Infused FRα-specific MOv19-27z CAR T cells mediated significant, albeit modest, reduction in tumor progression compared to the control mice treated with untransduced T cells (*p* = 0.01) or with anti-CD19 CAR T cells (*p* = 0.035), as measured by caliper-based tumor size (Fig. 4b).

We next measured the persistence of the transferred human T cells in vivo to determine whether modest antitumor activity was associated with poor T cell engraftment. Peripheral blood was obtained from MDA-231-bearing NSG mice treated with IV injections of T cells on day 60, 14 days after the last dose of transferred T cells, and quantified for the presence of human CD4+ and CD8+ T cells. Mice that received FRα-specific MOv19-27z T cells had readily detectable circulating human CD4+ and CD8+ T cells with significantly higher cell counts than those observed in mice treated with anti-CD19 CAR or untransduced T cells (Fig. 4c; *p =* 0.008 and *p* = 0.002, respectively), indicating that tumor antigen recognition drives the survival of the adoptively transferred FRα-specific CAR T cells in vivo*.*

We next assayed for FRα protein expression on residual tumors after treatment with untransduced, anti-CD19 CAR or FRα-specific CAR T cells, to determine whether tumor outgrowth in FRα CAR-treated mice was a byproduct of immune pressure and selective growth of FRα-negative cancer cells. MDA-231 tumors retained a stable FRα expression profile (Additional file 2: Figure S2), indicating that antigen loss did not occur after CAR T cell treatment in this TNBC xenograft model.

### FRα CAR T cells preferentially kill antigen overexpressing tumor cells

**Fig. 5:**
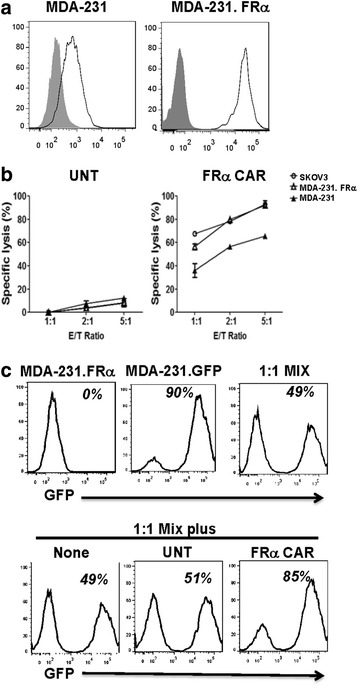
FRα CAR T cells preferentially lyse tumor cells overexpressing FRα. **a** The MDA-231 cell line was engineered to overexpress FRα (MDA-231.FRα). **b** FRα CAR T cells T cells lyse MDA-231.FRα and SKOV3 more efficiently compared to MDA-231 cells. **c** MDA-231 cells were engineered to express ~90 % GFP (MDA-231.GFP). MDA-231.GFP cells were then mixed with MDA-231.FRα cells at a 1:1 ratio (~50 % GFP expression). The mixed cells were treated with either UNT or FRα CAR T cells at a 3:1 of E/T ratio for 24 h. UNT T cell treatment had no impact on the mixed tumor cell (~50 % GFP expression), whereas treatment with FRα CAR T cells increased the number of GFP (+)-engineered tumor cells (~85 % GFP expression)

We previously showed that FRα CAR T cells can eradicate FRα^pos^ ovarian cancer xenografts in vivo [9, 10] and that FRα expression on MDA-231 cells is sufficient for recognition by FRα CAR T cells in vitro and in vivo. In parallel assays, we observed superior tumor regression in a FRα^pos^ SKOV3 ovarian cancer xenograft model where mice were treated with the same FRα CAR T cells (Additional file 3: Figure S3), reflecting a disparity in the killing of TNBC MDA-231 tumors versus FRα^pos^ SKOV3 ovarian tumors in vivo. We postulated that this resulted from the higher relative expression of FRα protein by SKOV3 than MDA-231, which reduced the potential of CAR T cells to exert their full function against TNBC in vivo and that TNBC cells with higher FRα expression might be more responsive to therapy. To test this hypothesis, MDA-231 cells were engineered to overexpress human FRα (referred to as MDA-231.FRα). The MDA-231.FRα cell line exhibited a 19**-**fold increase in mean fluorescence intensity for FRα expression compared to the parental line (Fig. 5a). In an in vitro cytotoxicity assay, FRα CAR T cells were capable of killing MDA-231.FRα cells as well as SKOV3 cells and more efficiently than parental MDA-231 cells (Fig. 5b). Untransduced T cells served as a negative control and did not recognize or kill tumor cells despite the high levels of FRα expression. Thus, it appears that tumor cells expressing antigen at low levels are less sensitive to killing by CAR T cells. We therefore sought to determine whether CAR T cells preferentially kill tumor cells that express antigen at higher levels and selectively spare tumor cells with lower FRα levels. We first engineered parental MDA-231 cells that express moderate levels of FRα to express green fluorescent protein (GFP), referred to as MDA-231.GFP, and then co-cultured MDA-231.FRα (GFP-negative) cells with MDA-231.GFP cells at a 1:1 ratio, where ~50 % of all MDA-231 cells were found to express GFP (Fig. 5c). The 1:1 MDA-231 mixture was then exposed to either untransduced or FRα-specific CAR T cells at a target to effector cell ratio of 1:3 for 24 h. Treatment with FRα CAR T cells resulted in a relative increase in the number of MDA-231.GFP-engineered tumor cells (~85 % GFP^pos^), reflecting a selective killing of the GFP-negative, FRα overexpressing MDA-231.FRα cell population (Fig. 5c). As anticipated, untransduced T cell treatment had no impact when co-cultured with tumor cells with ~50 % of MDA-231 cells still expressing GFP. These results suggest that tumor cells expressing higher antigen levels are preferentially killed, relative to those with lower target antigen expression.

### FRα CAR T cells induce rapid tumor regression of TNBC overexpressing FRα in vivo

**Fig. 6:**
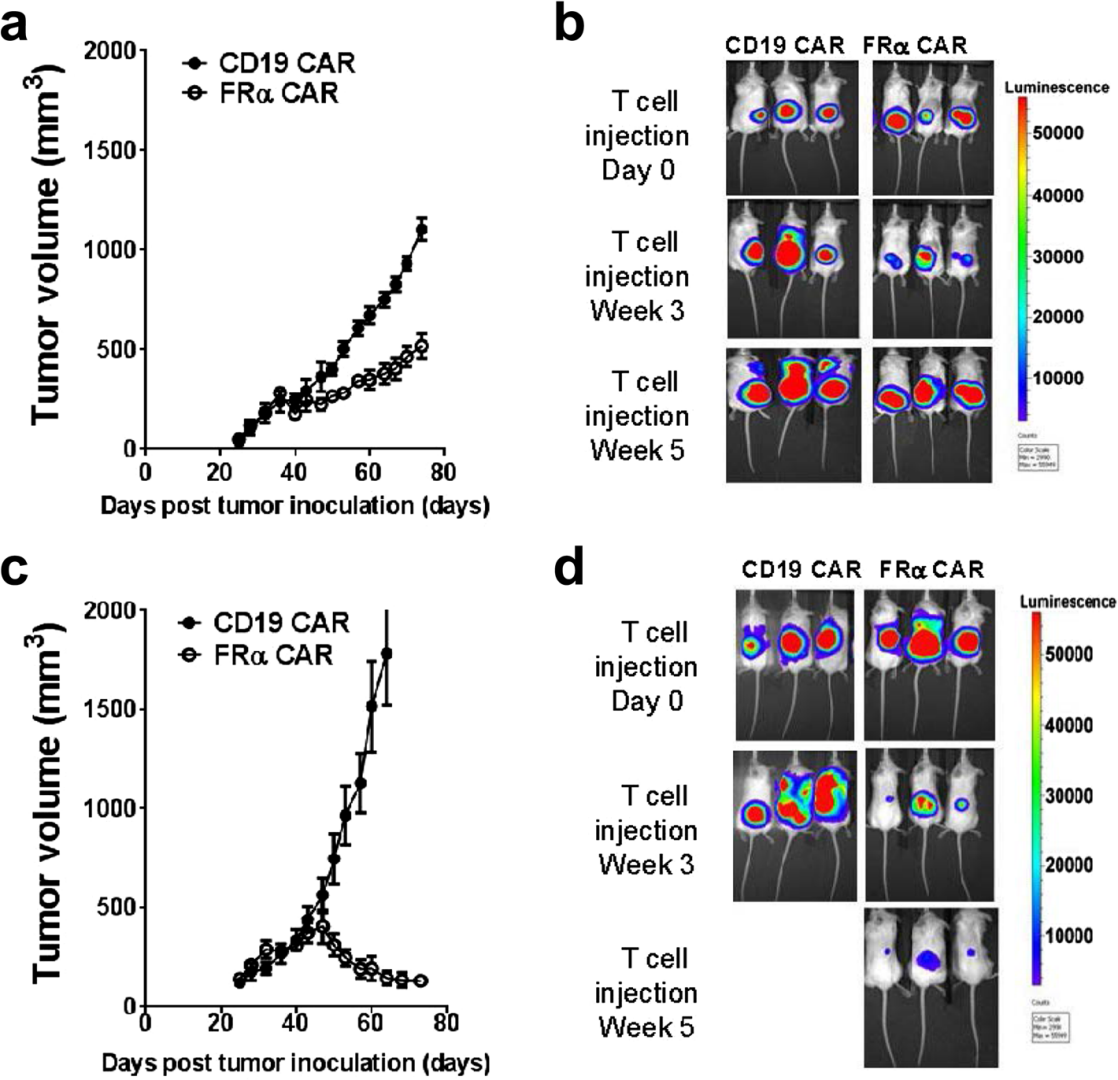
FRα CAR T cells induce rapid tumor regression of MDA-231.FRα in vivo*.* **a**-**d** NSG mice were inoculated with MDA-231 (**a**, **b**) or MDA.231.FRα tumor cells (**c**, **d**). Mice bearing established MDA-231.FRα or MDA-231 tumors received tail vein injections of 1 × 10^7^ CAR+ T cells on days 40 and 46, and tumor growth was monitored by caliper measurements (**a**, **c**) and BLI (**b**, **d**)

To evaluate the impact of the antigen level on antitumor activity of FRα CAR T cells in vivo, we inoculated NSG mice with MDA-231 or MDA-231.FRα cells and allowed for tumor growth. After 40 days, MDA-231.FRα tumors were modestly larger than their parental MDA-231 cells in mice, analogous to the known association between FRα overexpression and tumor progression [15] and the reported impact of FRα overexpression on ovarian cancer cell proliferation, migration, and invasion [15]. Mice-bearing established MDA-231.FRα or parental MDA-231 tumors received tail vein injections of 10^7^ CAR+ T cells on days 40 and 46, and tumor growth was monitored using caliper measurements and BLI. Consistent with our initial in vivo assays, FRα CAR T cells modestly delayed MDA-231 tumor growth (Fig. 6a, b). However, the same dose of FRα CAR T cells mediated more effective tumor regression in mice with MDA-231.FRα tumors, despite larger initial tumor burden (Fig. 6c, d). On days 60 and 74 after tumor inoculation, the average MDA-231.FRα tumor volume had decreased by 36 and 58 %, respectively, while the average MDA-231 tumor volume increased by 107 and 214 %, respectively (Additional file 4: Figure S4). By comparison, tumor volume increased sharply after treatment with anti-CD19 CAR T cells (Additional file 4: Figure S4). BLI confirmed that after treatment with FRα CAR T cells, mice bearing MDA-231.FRα tumors had less residual tumor burden, compared to mice with MDA-231 tumor (Fig. 6b, d), despite beginning therapy with greater tumor burden (Fig. 6b, d). Anti-CD19 CAR T cells had no antitumor activity against MDA-231 or MDA-231.FRα tumors in vivo (Fig. 6a, c). These results suggest that the regression of TNBC mediated by CAR T cells is dependent on a sufficient level of surface tumor antigen expression.

## Discussion

Although triple-negative breast cancer (TNBC) comprises approximately 15 % of all breast cancer subtypes, its aggressive biology and lack of specific systemic regimen or targeted therapy mandates the search for novel treatments specific for TNBC. Folate receptor-alpha (FRα; FOLR1) is a glycosyl-phosphatidyl inositol (GPI)-anchored protein that is overexpressed at both the protein and mRNA levels in TNBC [16], where it serves a biological role in TNBC cell growth and folate uptake. Strong FRα immunohistochemical (IHC) staining is highly associated with poor outcome in breast cancer patients [17], and while approximately 30 % of breast cancers express FRα, 70–80 % of stage IV metastatic TNBC tumors express FRα across multiple subtypes [4]. Importantly, the increased expression of FRα is associated with a significantly worse clinical outcome in TNBC [18]. Thus, the rationale for targeting FRα in TNBC is sound. Interestingly, endogenous immune responses against FRα are evident in cancer patients. For instance, Knutson et al. [19] demonstrated that immunity to FRα is prevalent in patients with breast and ovarian cancers where FRα-reactive lymphocytes having been identified in patients with these cancers and attempts to bolster this natural immunity are being investigated in preclinical and ongoing clinical trials in ovarian cancer and lung cancer [20, 21]. This led to our hypothesis that TNBC could be effectively targeted with immune-based cancer treatment using CAR T cells, an approach that has shown great utility on the treatment of hematological malignancy [22]. CAR T cell therapy represents a rapid approach to generate and apply a large number of antigen-specific T cells for the treatment of cancer, and in previous studies, we developed FRα-specific CAR T cells that demonstrated potent effects on human ovarian cancer in preclinical models [9, 10]. We therefore sought to utilize FRα CAR T cells to target TNBC.

Consistent with IHC assay results reported by others [4, 17, 18], we found positive surface expression of FRα protein on all (5/5) human TNBC cell lines tested by flow cytometry. FRα expression was largely moderate on these TNBC lines; however, IHC findings demonstrate that FRα protein can be expressed at high, intermediate, or low levels in TNBC, and as with primary breast tumors, the abundance of FRα mRNA varies among TNBC cell lines, with a subset expressing high levels [4]. As we showed earlier, FRα-specific CAR T cells are sensitive in vitro to FRα protein displayed on the cancer cell surface at a wide range of levels [9, 10]. In the current study, primary human T cells expressing a FRα-specific CAR recognized all human TNBC cell lines expressing FRα at different levels. FRα-specific T cells exhibited polyfunctionality in their ability to secrete IFN-γ, TNF-α, and IL-2 upon stimulation with FRα+ tumor cells. FRα CAR T cells also displayed potent cytolytic capacity in vitro against FRα+ TNBC cell lines. These in vitro tumor killing findings further support the notion that FRα has promise as a novel immunotherapy target for TNBC, which currently lacks effective targeted therapy. Importantly, two injections of FRα CAR T cells exhibited in vivo antitumor effects in a highly invasive and metastatic MDA-MB-231 xenograft model of human TNBC. Given the natural range of FRα protein expression level in TNBC, we postulated that the response to CAR T cell therapy may be more pronounced in patients with tumors expressing higher levels of FRα. Indeed, our preclinical studies show that the antitumor activity of FRα CAR T cells correlates with the level of surface antigen expressed by tumor cells, as demonstrated by the more dramatic tumor regressions mediated by CAR T cells in mice bearing MDA-231 TNBC tumors in which FRα was expressed at a high level.

Our results rationalize the application of FRα CAR T cell therapy in patients with TNBC whose tumors express FRα, particularly at a high level. It is notable that the FRα gene is positively regulated by the glucocorticoid receptor agonist, dexamethasone, at the transcriptional (P4 promoter) level and this profound regulation is further potentiated by inhibiting histone deacetylase (HDAC) [23]. This observation supports the notion that the efficacy of FRα CAR T cell therapy in TNBC patients with low to intermediate tumoral FRα levels may be improved in combination with dexamethasone and HDAC inhibitors that enhance FRα gene expression; however, additional preclinical studies of cell surface expression of FRα would be required to confirm this finding in vitro and in vivo, as well as their effects on normal tissue organ expression*.*

As an alternative approach to improve CAR T cell therapy for TNBC, simultaneous co-targeting of two different tumor-associated antigens may be applied to broaden the immune response and induce tumor elimination. For instance, like FRα, mesothelin, another GPI-linked cell surface glycoprotein present on mesothelial cells, is overexpressed in TNBC and has been shown to be an attractive immunotherapy target for CAR T cells [24]. We previously constructed a fully human anti-mesothelin CAR and showed that CAR-redirected T cells efficiently kill mesothelin-expressing tumors in vitro and in vivo [25]. Therefore, co-administration of FRα CAR T cells and mesothelin CAR T cells may provide superior antitumor effects in TNBC and also address potential issues of tumor antigen heterogeneity and antigen loss, which has been previously reported [26, 27]. In this line, we have previously established proof of principle for the application of bispecific CAR T cells with specificity for both mesothelin and FRα [28]. However, Anurathapan et al. [29] demonstrated optimal antitumor effects using two different antigen-specific CARs simultaneously, though this was insufficient to achieve a complete response. Small numbers of residual tumor cells were observed that appeared to express the targeted antigens and were resistant to repetitive T cell treatment, likely due to the low level of antigen expression determining tumor susceptibility to CAR T cells. Similarly, in a multicenter trial [30] of a novel FRα-targeted agent (EC145) in advanced, FRα-positive adenocarcinoma of the lung, there was an overall survival advantage and superior clinical benefit response for patients who had high expression of FRα on their tumors compared with those who had tumors with intermediate FRα expression. Based on these results, a phase II trial (clinicaltrials.gov/NCT01577654) evaluating the activity of EC145 versus EC145 plus docetaxel versus docetaxel alone in FRα high tumors has been initiated. Collectively, results from these studies suggest that pre-selection of patients with a high-level antigen expression could improve clinical response rates.

In the clinic, another T cell-based immunotherapy approach utilizing bispecific T cell engager (BiTE) is also under investigation [31, 32]. BiTEs provide a conventional drug approach in terms of storage, dosage, and delivery system; however, BiTEs have a short half-life and have to be given as a continuous infusion, which can be associated with systemic toxicities [33]. In addition, BiTEs lack active biodistribution once infused and may not penetrate tissue planes [33]. Compared with the BiTE approach, CAR T cell therapy has several advantages for TNBC and other cancers. CAR T cells can recognize and lyse cells bearing a very low level of target antigen, and CAR T cells can persist long term in vivo. Since our human FRα CAR T cells do not cross-react against mouse FRα protein, the potential for toxicity of this approach in the TNBC mouse model cannot be determined. Importantly, however, various strategies are being developed to create safer, regulatable CARs that control and/or minimize potential toxicity [34, 35].

While increased expression of FRα is associated with a poor clinical outcome in TNBC [17], FRα expression may vary by TNBC subtype and be associated with disease stage. Future studies will be required to determine the minimal and maximal threshold of FRα expression for activation and effective lysis by FRα CAR T cells upon stimulation with the TNBC cell lines or autologous tumor cells. Such results might aid in determining which patients may best benefit from FRα CAR T cell therapy and help determine the potential for off-tumor targeting of healthy tissues that may express low levels of antigen.

## Conclusion

We report the first demonstration of an in vivo antitumor response against established human TNBCxenografts using FRα-redirected CAR T cell therapy, with increased sensitivity observed against tumors bearing higher FRα protein levels. We therefore conlude that pateints with TNBC that expresses a high level of FRα protein may benefit from FRα-redirected CAR T cell therapy. More so, our preclinical studies serve to further accelerate the translation of FRα-targeted immunotherapies, including FRα CAR T cell therapy, to the clinic for TNBC.

## Additional files

Additional file 1: Figure S1. Expression of FRα in enzymatically digested MDA-231 and cell lines and correlation with IFN-γ secretion. FRα expression was determined using BD Quanti-Brite beads. The number of receptors per cell was calculated used at a 1:1 PE/protein ratio for the quantitative analysis of surface FRα expression. FRα antigen number per tumor cell was calculated by comparing the mean fluorescence intensity (MFI) versus the number of known PE molecules per bead. T47D and MDA-231 exhibited the highest number of FRα receptors per cell and AE17 mouse mesothelioma cell line displaying the lowest number of receptors per cell (A). Using a linear regression fitted line, the correlation between the Frα receptors per cell and IFN-γ secretion was calculated (B). (TIF 51 kb) Additional file 2: Figure S2. MDA-231 tumors retained a stable FRα expression profile after UNT, CD19 CAR, or FRα CAR T cell treatment. On day 73, mice bearing MDA-231 tumors were sacrificed and tumors were collected and cut up in RPMI 1640, washed, and centrifuged at room temperature at 800 rpm for 5 min and then resuspended in enzymatic digestion buffer (collagenase [0.2 mg/mL] and DNase [30 units/mL] in RPMI 1640) for overnight digestion at room temperature. FRα-specific mAb MOv18 was used to measure FRα expression on MDA-231 tumors treated with different T cells. (TIF 51 kb) Additional file 3: Figure S3. FRα CAR T cells induced SKOV3 ovarian tumor rapid regression in vivo*.* NSG mice were inoculated with SKOV3 ovarian cancer tumor cells. Mice bearing established SKOV3 tumors received tail vein injections of 10^7^ CAR+ T cells on days 40 and 46 and tumor growth was monitored by caliper measurements. (TIF 140 kb) Additional file 4: Figure S4. Tumor volume fold changes after CAR T cell treatment on days 60 and 74. NSG mice were inoculated with MDA-231 or MDA-231. FRα tumor cells. Mice bearing established MDA-231.FRα or MDA-231 tumors received tail vein injections of 1 × 10^7^ CAR+ T cells on days 40 and 46, and tumor growth was monitored by caliper measurements. (TIF 37 kb)
