# Supplementary figures and images for: Effective adoptive immunotherapy of triple-negative breast cancer by folate receptor-alpha redirected CAR T cells is influenced by surface antigen expression level
Source: J Hematol Oncol. 2016 Jul 20;9:56. doi: 10.1186/s13045-016-0285-y (PMC4955216; doi:10.1186/s13045-016-0285-y)

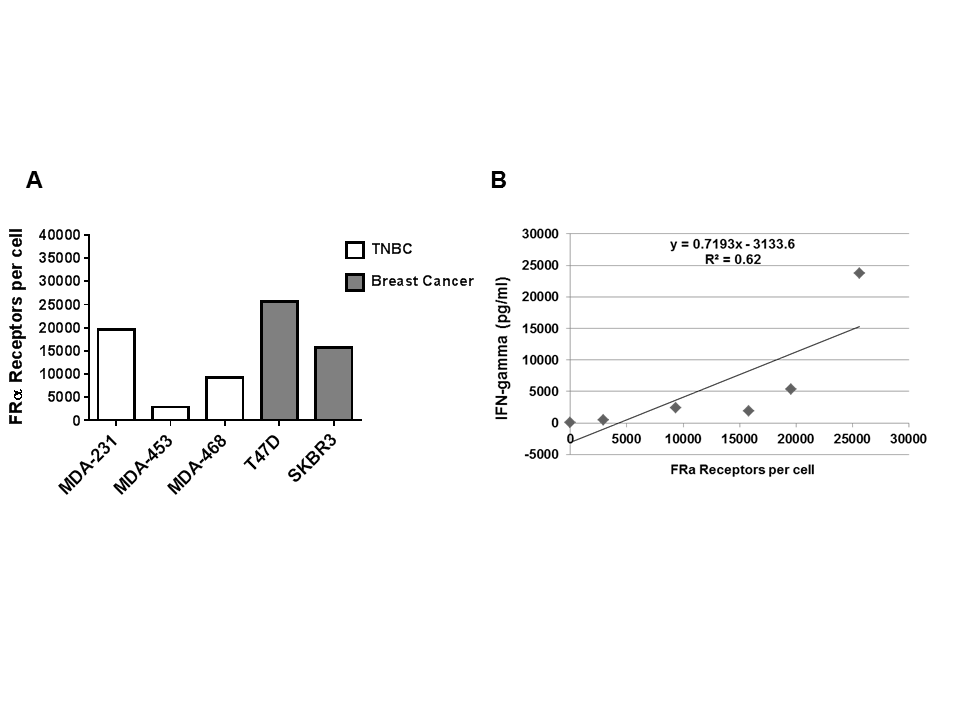

Supplement: Additional file 1: Figure S1. — Expression of FRα in enzymatically digested MDA-231 and cell lines and correlation with IFN-γ secretion. FRα expression was determined using BD Quanti-Brite beads. The number of receptors per cell was calculated used at a 1:1 PE/protein ratio for the quantitative analysis of surface FRα expression. FRα antigen number per tumor cell was calculated by comparing the mean fluorescence intensity (MFI) versus the number of known PE molecules per bead. T47D and MDA-231 exhibited the highest number of FRα receptors per cell and AE17 mouse mesothelioma cell line displaying the lowest number of receptors per cell (A). Using a linear regression fitted line, the correlation between the Frα receptors per cell and IFN-γ secretion was calculated (B). (TIF 51 kb) [file 13045_2016_285_MOESM1_ESM.tif]

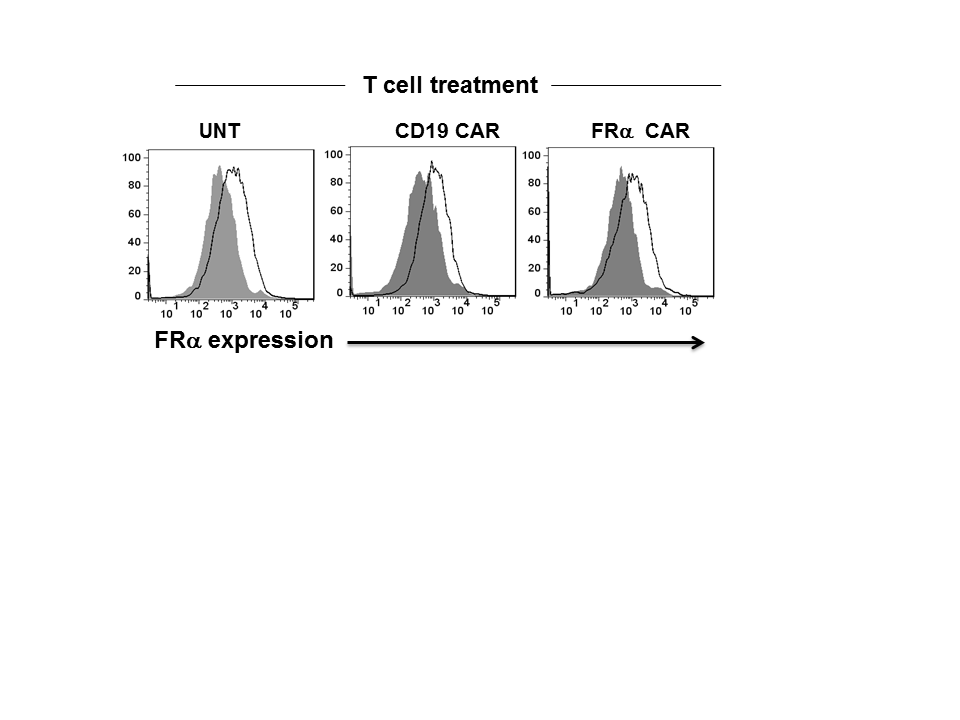

Supplement: Additional file 2: Figure S2. — MDA-231 tumors retained a stable FRα expression profile after UNT, CD19 CAR, or FRα CAR T cell treatment. On day 73, mice bearing MDA-231 tumors were sacrificed and tumors were collected and cut up in RPMI 1640, washed, and centrifuged at room temperature at 800 rpm for 5 min and then resuspended in enzymatic digestion buffer (collagenase [0.2 mg/mL] and DNase [30 units/mL] in RPMI 1640) for overnight digestion at room temperature. FRα-specific mAb MOv18 was used to measure FRα expression on MDA-231 tumors treated with different T cells. (TIF 51 kb) [file 13045_2016_285_MOESM2_ESM.tif]

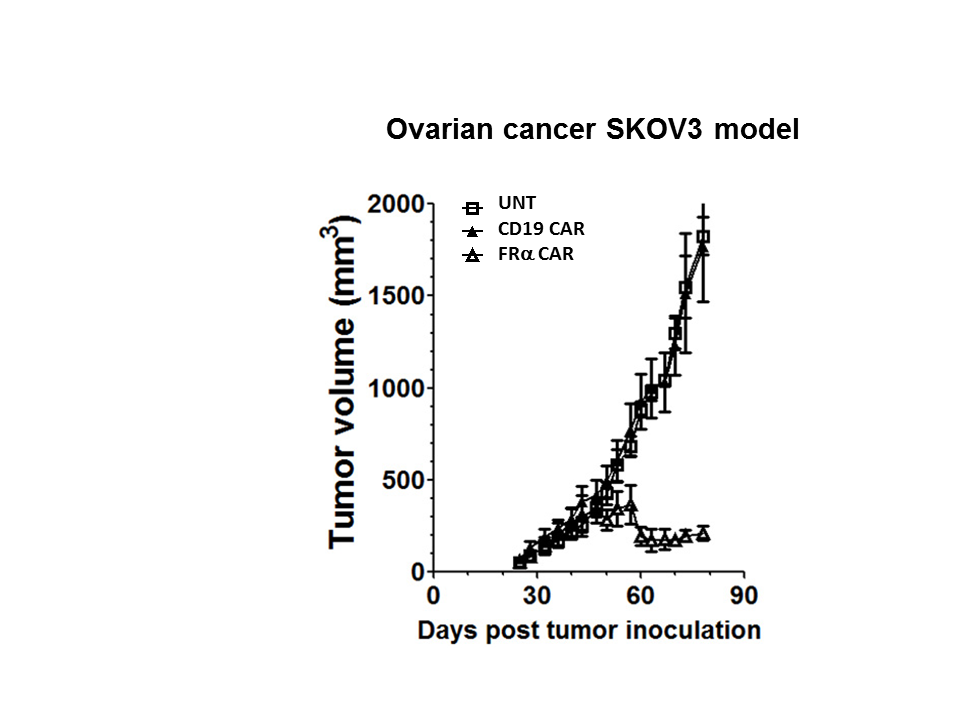

Supplement: Additional file 3: Figure S3. — FRα CAR T cells induced SKOV3 ovarian tumor rapid regression in vivo. NSG mice were inoculated with SKOV3 ovarian cancer tumor cells. Mice bearing established SKOV3 tumors received tail vein injections of 107 CAR+ T cells on days 40 and 46 and tumor growth was monitored by caliper measurements. (TIF 140 kb) [file 13045_2016_285_MOESM3_ESM.tif]

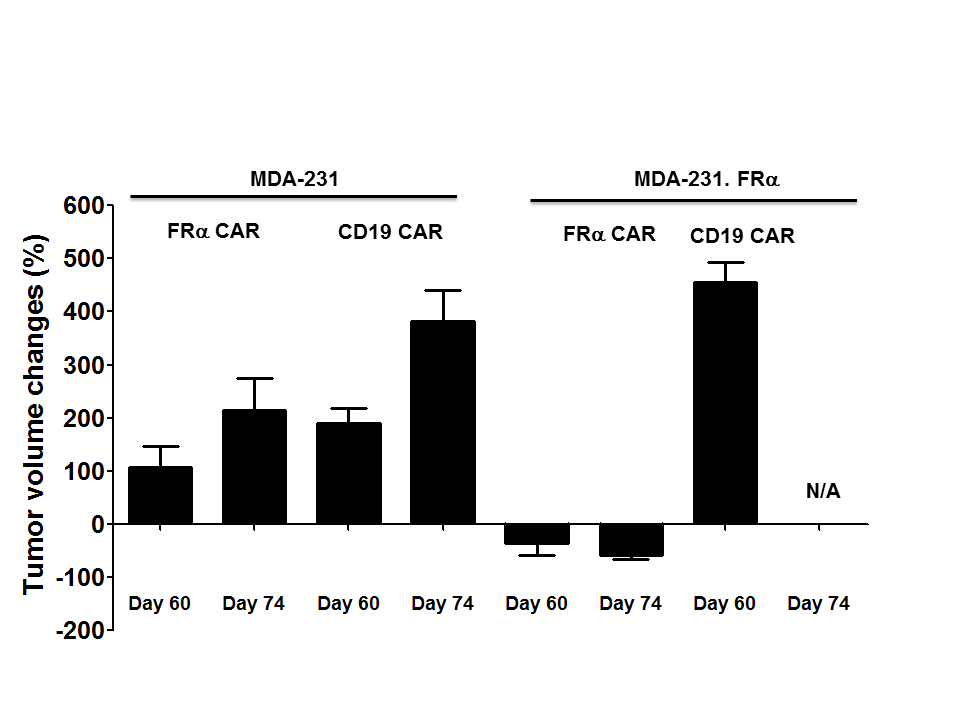

Supplement: Additional file 4: Figure S4. — Tumor volume fold changes after CAR T cell treatment on days 60 and 74. NSG mice were inoculated with MDA-231 or MDA-231. FRα tumor cells. Mice bearing established MDA-231.FRα or MDA-231 tumors received tail vein injections of 1 × 10^7^ CAR+ T cells on days 40 and 46, and tumor growth was monitored by caliper measurements. (TIF 37 kb) [file 13045_2016_285_MOESM4_ESM.tif]
